# Supplementary material for: Gene expression composite scores of cellular senescence predict aging health outcomes in the Health and Retirement Study
Source: Nat Commun. 2025 Oct 10;16:9044. doi: 10.1038/s41467-025-64835-8 (PMC12514264; doi:10.1038/s41467-025-64835-8)
Supplement: Supplementary file 1 — Supplementary Information [file 41467_2025_64835_MOESM1_ESM.pdf]

Supplementary Information

Supplementary Table 1. Comparison of the Expression Level of Genes from Dehkordi et al. Lists in Blood and Brain in Human Protein Atlas

List	Gene	Brain	Blood	List	Gene	Brain	Blood	List	Gene	Brain	Blood
CSP	*CDKN2D*	7	8.1	SIP	*SOD1*	9.2	7.4	SRP	*IGFBP7*	7.2	5.4
CSP	*ETS2*	5.6	7.4	SIP	*MAP2K1*	6.6	5.3	SRP	*VIM*	8.2	10.5
CSP	*RB1*	3.8	4.3	SIP	*GSK3B*	4.4	4	SRP	*FN1*	5.1	-0.7
CSP	*E2F3*	2.4	4.3	SIP	*PIK3CA*	3	3.6	SRP	*SPARC*	9.9	5
CSP	*CDK6*	1.1	4.2	SIP	*SOD2*	5.8	10.1	SRP	*IGFBP4*	4.6	1.3
CSP	*RBL2*	4	4.3	SIP	*MAPK14*	3.8	6.5	SRP	*TIMP1*	6.4	6.9
CSP	*ATM*	2.3	3.6	SIP	*IGF1R*	2.8	3.4	SRP	*TBX2*	2.1	-6.6
CSP	*BMI1*	4.8	4.4	SIP	*TP53BP1*	4.1	3.5	SRP	*TBX3*	1.4	-6.6
CSP	*MDM2*	3.4	5	SIP	*NBN*	3.5	4.1	SRP	*COL1A1*	2.4	2.1
CSP	*CDK4*	4.6	6.2	SIP	*HRAS*	6.5	1.5	SRP	*COL3A1*	2.7	0.1
CSP	*CCNE1*	1.4	5.1	SIP	*CITED2*	4.9	8.3	SRP	*IGFBP2*	4.5	4.4
CSP	*E2F1*	2.8	5.1	SIP	*CREG1*	5.5	7	SRP	*TGFB1I1*	2.2	-0.2
CSP	*CHEK2*	-0.5	4	SIP	*ABL1*	4.3	4.2	SRP	*PTEN*	4.1	4.1
CSP	*CHEK1*	0.1	4.7	SIP	*MORC3*	3.1	5.1	SRP	*CD44*	4.9	8.2
CSP	*CDKN1A*	5.1	6.2	SIP	*NFKB1*	2.3	4.6	SRP	*NFIA*	3.4	3.4
CSP	*TWIST1*	0.1	-6.6	SIP	*AKT1*	5.7	5.6	SRP	*CALR*	7.1	9.1
CSP	*CCND1*	3.7	0.6	SIP	*CDKN1B*	5.5	6.1	SRP	*TIMP2*	6.9	4.6
CSP	*ETS1*	3.5	4.7	SIP	*EGR1*	6.1	7.4	SRP	*CXCL8*	2	11.6
CSP	*TP53*	3.7	4.3	SIP	*RBL1*	0.5	3.8	SRP	*IL6*	2	5.5
CSP	*CDKN2A*	1.3	0.4	SIP	*MAP2K6*	1.5	4	SRP	*FGF2*	3.4	-3.3
CSP	*CDK2*	1.3	4.7	SIP	*IGF1*	0.7	-0.3	SRP	*FGF7*	1.9	-3.3
CSP	*SATB1*	5.2	5.3	SIP	*IRF3*	4.6	4.6	SRP	*AKT1*	5.7	5.6
-	-	-	-	SIP	*PCNA*	4.6	8.6	SRP	*CXCL2*	2.1	6.4
-	-	-	-	SIP	*GADD45A*	5	7.1	SRP	*VEGFA*	4.4	5.5
-	-	-	-	SIP	*MAP2K3*	3.4	7.7	SRP	*CXCL1*	2.2	5.4
-	-	-	-	SIP	*IGFBP5*	5.2	-0.5	SRP	*PLAUR*	2.4	9.4
-	-	-	-	SIP	*SIRT1*	3.1	4.3	SRP	*SERPINE1*	2.8	0.7
-	-	-	-	SIP	*ING1*	3.4	4.3	SRP	*LMNB1*	1.1	5.1
-	-	-	-	SIP	*TGFB1*	3.1	6.4	SRP	*GLB1*	3.5	4.4
-	-	-	-	SIP	*TERF2*	4.7	4.2	SRP	*VEGFB*	7	2
-	-	-	-	SIP	*CCNB1*	2.5	5.7	SRP	*CCL2*	5.6	6.6
-	-	-	-	SIP	*PRKCD*	3.7	6.2	SRP	*IL1B*	3.5	7.6
-	-	-	-	SIP	*CDC25C*	-2.3	2.5	SRP	*CXCL5*	0.7	0.3
-	-	-	-	SIP	*IGFBP3*	2.9	0.3	SRP	*SERPINB2*	-1.3	5.2
-	-	-	-	SIP	*ALDH1A3*	2.4	-1	SRP	*IL11*	0	-0.3
-	-	-	-	SIP	*MYC*	3.6	5.7	SRP	*IL1A*	0	0
-	-	-	-	SIP	*NOX4*	-1	-1.7	SRP	*CCL5*	1.2	6.9
-	-	-	-	SIP	*CCNA2*	0.1	5.3	SRP	*TNF*	0	4.4
-	-	-	-	SIP	*CDKN2C*	3.1	5.1	SRP	*CCL20*	-0.5	2.8
-	-	-	-	SIP	*TERT*	-6.6	-6.6	SRP	*MMP1*	-1	-1.7
-	-	-	-	SIP	*ID1*	5.6	3.2	SRP	*CCL8*	1.5	0.8
-	-	-	-	SIP	*IGFBP7*	7.2	5.4	SRP	*MMP3*	-0.5	-6.6
-	-	-	-	SIP	*CDKN1C*	3.9	0.8	SRP	*MMP12*	-6.6	-6.6
-	-	-	-	SIP	*IRF7*	1.5	0.7	SRP	*MMP10*	-2.3	-3.3
-	-	-	-	SIP	*IFNG*	-6.6	3.5	-	-	-	-
-	-	-	-	SIP	*CDKN2B*	0.6	-1.3	-	-	-	-
-	-	-	-	SIP	*PLAU*	1.5	5.2	-	-	-	-
-	-	-	-	SIP	*IRF5*	1.1	3.9	-	-	-	-

Note

CSP – Canonical Senescence Pathway Score; SIP – Senescence Initiating Pathway Score; SRP – Senescence Response Pathway Score;

Expression levels are indicated by log2 transformed normalized expression (nTPM) values derived from information on the open-access online source Human Protein Atlas (https://www.proteinatlas.org/), which documents the RNA expression of each gene across different organs. Specifically, the expression level in the brain is based on cerebral cortex, and the expression level in blood is based on bone marrow.

Supplementary Table 2. Regressions of Aging-Related Health Outcomes on Senescence Scores

	CSP		SIP		SRP		Sum Score		SenMayo		
Outcome	beta/OR	p	beta/OR	p	beta/OR	p	beta/OR	p	beta/OR	p	N
PC GrimAge AA	0.00	0.920	0.31***	0.000	0.25***	0.000	0.29***	0.000	0.24***	0.000	3,580
PhenoAge AA	0.03	0.445	0.26***	0.000	0.21***	0.000	0.25***	0.000	0.21***	0.000	3,580
DunedinPACE	0.04	0.108	0.26***	0.000	0.19***	0.000	0.24***	0.000	0.16***	0.000	3,580
ExpBioAge AA	-0.01	0.692	0.23***	0.000	0.16***	0.000	0.20***	0.000	0.13***	0.000	2,660
Multimorbidity	0.00	0.925	0.15***	0.000	0.09***	0.000	0.12***	0.000	0.07***	0.001	3,580
Cognitive Function	-0.03	0.094	-0.07***	0.001	-0.05*	0.016	-0.07***	0.001	-0.05*	0.019	3,580
6yr Mortality	1.01	0.930	1.67***	0.000	1.38***	0.000	1.52***	0.000	1.32***	0.000	3,554

Note
* p<0.05, ** p<0.01, *** p<0.001
CSP – Canonical Senescence Pathway Score; SIP – Senescence Initiating Pathway Score; SRP – Senescence Response Pathway Score; Sum Score – Senescence Summary Score; SenMayo – SenMayo Score; ExpBioAge – Expanded Biological Age; AA – Age Acceleration; PC – Principal Component.
All models are adjusted for all covariates (age, sex, race/ethnicity, education, BMI categories, smoking, drinking, and insomnia symptoms) and batch/plate.
When mortality is the outcome, odds ratios are reported, and senescence scores are standardized. Standardized coefficients are reported for the other outcomes. For the models predicting mortality, N=3,554; For the models predicting ExpBioAgeAA, N=2,660; For all other models, N=3,580.

Supplementary Table 3. Regressions of Aging-Related Health Outcomes on Senescence Scores Adjusted for DunedinPACE

	CSP		SIP		SRP		Sum Score		SenMayo		
Outcome	beta/OR	p	beta/OR	p	beta/OR	p	beta/OR	p	beta/OR	p	N
ExpBioAge AA	-0.02	0.410	0.16***	0.000	0.11***	0.000	0.13***	0.000	0.08**	0.002	2,660
Multimorbidity	-0.01	0.736	0.09***	0.000	0.04*	0.029	0.07**	0.004	0.03	0.097	3,580
Cognitive Function	-0.03	0.121	-0.06**	0.006	-0.04	0.066	-0.05**	0.008	-0.04	0.061	3,580
6yr Mortality	0.97	0.674	1.43***	0.000	1.22**	0.004	1.30***	0.001	1.18*	0.021	3,554

Note
* p<0.05, ** p<0.01, *** p<0.001
CSP – Canonical Senescence Pathway Score; SIP – Senescence Initiating Pathway Score; SRP – Senescence Response Pathway Score; Sum Score – Senescence Summary Score; SenMayo – SenMayo Score; ExpBioAge – Expanded Biological Age; AA – Age Acceleration.
All models are adjusted for all covariates (age, sex, race/ethnicity, education, BMI categories, smoking, drinking, and insomnia symptoms) and batch/plate.
When mortality is the outcome, odds ratios are reported, and senescence scores and DunedinPACE are standardized for comparison. Standardized coefficients are reported for the other outcomes. For the models predicting mortality, N=3,554; For the models predicting ExpBioAgeAA, N=2,660; For all other models, N=3,580.

Supplementary Table 4. Relative Proportions of Age Groups across Racial/Ethnic Groups

Age Group	Race/Ethnicity	Proportion	95% Conf. Interval		Wald Test P-Value
Aged 55-64	NH White	38.4%	0.36	0.41	Ref
	NH Black	46.1%	0.41	0.52	**0.011**
	Hispanic	46.5%	0.40	0.53	**0.016**
	NH Others	57.4%	0.46	0.68	**0.001**
Aged 65-74	NH White	35.2%	0.33	0.38	Ref
	NH Black	36.3%	0.31	0.42	0.718
	Hispanic	34.8%	0.29	0.41	0.910
	NH Others	32.4%	0.23	0.44	0.603
Aged 75-84	NH White	18.9%	0.17	0.20	Ref
	NH Black	12.2%	0.10	0.15	**0.000**
	Hispanic	12.4%	0.10	0.16	**0.000**
	NH Others	7.5%	0.04	0.13	**0.000**
Aged 85+	NH White	7.6%	0.07	0.09	Ref
	NH Black	5.5%	0.03	0.09	0.180
	Hispanic	6.3%	0.04	0.10	0.425
	NH Others	2.7%	0.01	0.07	**0.001**

Note

NH – Non-Hispanic. For each racial/ethnic group, the percentages of all age groups add up to 100%.

Supplementary Table 5. Logistic Regressions of 6-Year Mortality on Senescence Scores - Comparison between with and without Respondents with no Contact in 2022

	CSP		SIP		SRP		Sum Score		SenMayo		
Outcome	OR	p	OR	p	OR	p	OR	p	OR	p	N
Main Model											
6yr Mortality	1.01	0.930	1.67***	0.000	1.38***	0.000	1.52***	0.000	1.32***	0.000	3,554
6yr Mortality DunedinPACE Adj	0.97	0.674	1.43***	0.000	1.22**	0.004	1.30***	0.001	1.18*	0.021	3,554
Excluding No Contacts in 2022											
6yr Mortality	0.99	0.938	1.74***	0.000	1.37***	0.000	1.53***	0.000	1.33***	0.000	3,150
6yr Mortality DunedinPACE Adj	0.96	0.531	1.46***	0.000	1.21**	0.009	1.30**	0.001	1.19*	0.020	3,150

Note

* p<0.05, ** p<0.01, *** p<0.001

CSP – Canonical Senescence Pathway Score; SIP – Senescence Initiating Pathway Score; SRP – Senescence Response Pathway Score; Sum Score – Senescence Summary Score; SenMayo – SenMayo Score.

All models are adjusted for all covariates (age, sex, race/ethnicity, education, BMI categories, smoking, drinking, and insomnia symptoms) and batch/plate.

Senescence scores and DunedinPACE are standardized for comparison.

Supplementary Table 6. Results of the OLS Model Predicting the Senescence Scores Adjusted for Cell Composition (N=3,298)

N=3,298	CCA		MD		SASP		Sum Score		SenMayo	
	beta	p	beta	p	beta	p	beta	p	beta	p
**Age: Ref - Aged 55-64**										
Aged 65-74	-0.04	0.067	-0.02	0.339	0.01	0.510	-0.01	0.567	-0.02	0.311
Aged 75-84	-0.05**	0.006	0.00	0.970	0.04*	0.022	0.01	0.647	0.02	0.214
Aged 85+	-0.06**	0.001	-0.01	0.328	0.02	0.234	-0.01	0.451	0.01	0.512
**Female**	0.17***	0.000	0.04*	0.011	0.11***	0.000	0.11***	0.000	0.09***	0.000
**RE: Ref - Non-Hispanic White**										
Non-Hispanic Black	0.08***	0.000	0.05***	0.000	0.01	0.422	0.05***	0.001	0.00	0.716
Hispanic	-0.02	0.365	-0.02	0.204	-0.03	0.067	-0.02	0.082	-0.03	0.067
Non-Hispanic Other	0.01	0.727	-0.01	0.539	-0.02	0.255	-0.01	0.444	-0.01	0.500
**Education: Ref - Less than High School**										
High School	-0.02	0.461	-0.02	0.259	0.00	0.857	-0.02	0.370	0.00	0.983
Some College	-0.01	0.751	-0.03	0.126	-0.01	0.567	-0.02	0.235	0.00	0.931
College and Higher	-0.02	0.523	-0.05*	0.026	-0.02	0.353	-0.04	0.067	0.00	0.994
**BMI: Ref - Normal**										
Overweight	0.01	0.830	0.01	0.622	-0.01	0.538	0.00	0.919	-0.04*	0.021
Obese I	0.02	0.496	0.03	0.064	-0.02	0.330	0.01	0.508	-0.06***	0.000
Obese II	0.04	0.106	0.06***	0.001	0.03	0.073	0.06**	0.003	-0.03	0.062
**Cumulative Packs Smoked**	0.03	0.149	0.00	0.862	-0.03	0.065	-0.01	0.607	-0.03*	0.026
**Total Drinks Weekly**	0.04*	0.048	0.02	0.203	0.03	0.057	0.03*	0.046	0.05**	0.002
**Insomnia Symptoms**	0.02	0.371	0.00	0.785	-0.02	0.202	0.00	0.732	-0.02	0.126
**Adjusted R2**	0.23		0.59		0.46		0.51		0.53	

Note

* p<0.05, ** p<0.01, *** p<0.001

CSP – Canonical Senescence Pathway Score; SIP – Senescence Initiating Pathway Score; SRP – Senescence Response Pathway Score; Sum Score – Senescence Summary Score; SenMayo – SenMayo Score.

The model is adjusted for batch/plate and cell composition. Specifically, the percentages of granulocytes, natural killer cells, B cells, CD4 cells, CD8 cells, and monocytes are included in the models as covariates. Standardized coefficients are reported.

Supplementary Table 7. Regressions of Aging-Related Health Outcomes on Senescence Scores Adjusted for Cell Composition

	CSP		SIP		SRP		Sum Score		SenMayo		
Outcome	beta/OR	p	beta/OR	p	beta/OR	p	beta/OR	p	beta/OR	p	N
PC GrimAge AA	0.07***	0.000	0.09***	0.000	0.05*	0.014	0.09***	0.000	0.01	0.704	3,298
PhenoAge AA	0.06	0.058	0.11***	0.000	0.08**	0.002	0.12***	0.000	0.05	0.083	3,298
DunedinPACE	0.09***	0.000	0.13***	0.000	0.05*	0.017	0.11***	0.000	-0.01	0.637	3,298
ExpBioAge AA	0.04	0.145	0.06	0.121	0.01	0.804	0.04	0.182	-0.07*	0.023	2,596
Multimorbidity	0.02	0.332	0.12***	0.000	0.03	0.153	0.08**	0.003	0.01	0.741	3,298
Cognitive Function	-0.04	0.053	-0.06*	0.024	-0.03	0.218	-0.05*	0.028	-0.03	0.213	3,298
6yr Mortality	1.03	0.716	1.41***	0.001	1.15	0.076	1.26*	0.013	1.06	0.473	3,276

Note
* p<0.05, ** p<0.01, *** p<0.001
CSP – Canonical Senescence Pathway Score; SIP – Senescence Initiating Pathway Score; SRP – Senescence Response Pathway Score; Sum Score – Senescence Summary Score; SenMayo – SenMayo Score; ExpBioAge – Expanded Biological Age; AA – Age Acceleration; PC – Principal Component.
All models are adjusted for all covariates (age, sex, race/ethnicity, education, BMI categories, smoking, drinking, insomnia symptoms), batch/plate, and cell composition. Specifically, the percentages of granulocytes, natural killer cells, B cells, CD4 cells, CD8 cells, and monocytes are included in the models as covariates.
When mortality is the outcome, odds ratios are reported, and senescence scores are standardized. Standardized coefficients are reported for the other outcomes.

Supplementary Table 8. Regressions of Aging-Related Health Outcomes on Senescence Scores Adjusted for DunedinPACE and Cell Composition

	CSP		SIP		SRP		Sum Score		SenMayo		
Outcome	beta/OR	p	beta/OR	p	beta/OR	p	beta/OR	p	beta/OR	p	N
ExpBioAge AA	0.02	0.486	0.03	0.387	0.00	0.856	0.02	0.557	-0.06*	0.028	2,596
Multimorbidity	0.00	0.838	0.10***	0.001	0.02	0.329	0.06*	0.027	0.01	0.668	3,298
Cognitive Function	-0.03	0.083	-0.05*	0.042	-0.03	0.262	-0.05*	0.050	-0.03	0.204	3,298
6yr Mortality	0.97	0.667	1.32**	0.004	1.11	0.168	1.17	0.061	1.06	0.432	3,276

Note
* p<0.05, ** p<0.01, *** p<0.001
CSP – Canonical Senescence Pathway Score; SIP – Senescence Initiating Pathway Score; SRP – Senescence Response Pathway Score; Sum Score – Senescence Summary Score; SenMayo – SenMayo Score; ExpBioAge – Expanded Biological Age; AA – Age Acceleration; PC – Principal Component.
All models are adjusted for all covariates (age, sex, race/ethnicity, education, BMI categories, smoking, drinking, and insomnia symptoms), batch/plate, and cell composition. Specifically, the percentages of granulocytes, natural killer cells, B cells, CD4 cells, CD8 cells, and monocytes are included in the models as covariates.
When mortality is the outcome, odds ratios are reported, and senescence scores and DunedinPACE are standardized for comparison. Standardized coefficients are reported for the other outcomes.

Supplementary Table 9. Results of Ordinary Least Squares Regression Models Predicting the Senescence Scores Adjusted for False Discovery Rate (FDR)

N=3,580	CSP	SIP	SRP	Sum Score	SenMayo
**Age: Ref - Aged 55-64**					
Aged 65-74	-0.05	0.02	0.06*	0.03	0.04
Aged 75-84	-0.09***	0.07***	0.13***	0.08***	0.11***
Aged 85+	-0.09***	0.05**	0.10***	0.05***	0.10***
**Female**	0.20***	-0.03	0.02	0.04	0.01
**RE: Ref - Non-Hispanic White**					
Non-Hispanic Black	0.12***	-0.02	-0.07***	-0.02	-0.07***
Hispanic	-0.01	-0.04*	-0.06**	-0.05**	-0.05*
Non-Hispanic Other	0.01	-0.02	-0.04*	-0.03	-0.03
**Education: Ref - Less than High School**					
High School	-0.02	0.00	0.02	0.00	0.03
Some College	0.00	-0.02	0.00	-0.01	0.01
College and Higher	-0.02	-0.04	-0.01	-0.03	0.02
**BMI: Ref - Normal**					
Overweight	0.01	0.02	-0.01	0.01	-0.03
Obese I	0.02	0.04	-0.02	0.02	-0.05*
Obese II	0.02	0.09***	0.06*	0.08***	0.00
**Cumulative Packs Smoked**	0.02	0.02	-0.01	0.01	-0.01
**Total Drinks Weekly**	0.02	0.01	0.03	0.02	0.04
**Insomnia Symptoms**	0.01	0.02	0.00	0.01	-0.01
**Adjusted R2**	0.203	0.404	0.247	0.342	0.291

Note
* p<0.05, ** p<0.01, *** p<0.001
Stars indicate significance based on FDR-adjusted p values.
CSP – Canonical Senescence Pathway Score; SIP – Senescence Initiating Pathway Score; SRP – Senescence Response Pathway Score; Sum Score – Senescence Summary Score; SenMayo – SenMayo Score.

Supplementary Table 10. Regressions of Aging-Related Health Outcomes on Senescence Scores Adjusted for Cell Composition Adjusted for False Discovery Rate (FDR)

	CSP		SIP		SRP		Sum Score		SenMayo		
Outcome	beta/OR	p FDR	beta/OR	p FDR	beta/OR	p FDR	beta/OR	p FDR	beta/OR	p FDR	N
PC GrimAge AA	0.00	0.920	0.31***	0.000	0.25***	0.000	0.29***	0.000	0.24***	0.000	3,580
PhenoAge AA	0.03	0.445	0.26***	0.000	0.21***	0.000	0.25***	0.000	0.21***	0.000	3,580
DunedinPACE	0.04	0.108	0.26***	0.000	0.19***	0.000	0.24***	0.000	0.16***	0.000	3,580
ExpBioAge AA	-0.01	0.692	0.23***	0.000	0.16***	0.000	0.20***	0.000	0.13***	0.000	2,660
Multimorbidity	0.00	0.925	0.15***	0.000	0.09***	0.000	0.12***	0.000	0.07***	0.001	3,580
Cognitive Function	-0.03	0.094	-0.07***	0.001	-0.05*	0.016	-0.07***	0.001	-0.05*	0.019	3,580
6yr Mortality	1.01	0.930	1.67***	0.000	1.38***	0.000	1.52***	0.000	1.32***	0.000	3,554

Note
* p<0.05, ** p<0.01, *** p<0.001
Stars indicate significance based on FDR-adjusted p values.
CSP – Canonical Senescence Pathway Score; SIP – Senescence Initiating Pathway Score; SRP – Senescence Response Pathway Score; Sum Score – Senescence Summary Score; SenMayo – SenMayo Score; ExpBioAge – Expanded Biological Age; AA – Age Acceleration; PC – Principal Component.
All models are adjusted for all covariates (age, sex, race/ethnicity, education, BMI categories, smoking, drinking, and insomnia symptoms) and batch/plate.
When mortality is the outcome, odds ratios are reported, and senescence scores are standardized. Standardized coefficients are reported for the other outcomes. For the models predicting mortality, N=3,554; For the models predicting ExpBioAgeAA, N=2,660; For all other models, N=3,580.

Supplementary Table 11. Regressions of Aging-Related Health Outcomes on Senescence Scores Adjusted for DunedinPACE and Adjusted for False Discovery Rate (FDR)

	CSP		SIP		SRP		Sum Score		SenMayo		
Outcome	beta/OR	p FDR	beta/OR	p FDR	beta/OR	p FDR	beta/OR	p FDR	beta/OR	p FDR	N
ExpBioAge AA	-0.02	0.410	0.16***	0.000	0.11***	0.000	0.13***	0.000	0.08**	0.002	2,660
Multimorbidity	-0.01	0.736	0.09***	0.000	0.04*	0.029	0.07**	0.004	0.03	0.097	3,580
Cognitive Functioning	-0.03	0.121	-0.06**	0.006	-0.04	0.066	-0.05**	0.008	-0.04	0.061	3,580
6yr Mortality	0.97	0.674	1.43***	0.000	1.22**	0.004	1.30***	0.001	1.18*	0.021	3,554

Note
* p<0.05, ** p<0.01, *** p<0.001
Stars indicate significance based on FDR-adjusted p values.
CSP – Canonical Senescence Pathway Score; SIP – Senescence Initiating Pathway Score; SRP – Senescence Response Pathway Score; Sum Score – Senescence Summary Score; SenMayo – SenMayo Score; ExpBioAge – Expanded Biological Age; AA – Age Acceleration.
All models are adjusted for all covariates (age, sex, race/ethnicity, education, BMI categories, smoking, drinking, and insomnia symptoms) and batch/plate.
When mortality is the outcome, odds ratios are reported, and senescence scores and DunedinPACE are standardized for comparison. Standardized coefficients are reported for the other outcomes. For the models predicting mortality, N=3,554; For the models predicting ExpBioAgeAA, N=2,660; For all other models, N=3,580.

Supplementary Table 12. Regressions of Epigenetic Aging Measures on Senescence Scores

	PCGrimAgeAA	PhenoAgeAA	DunedinPACE	PCHorvath2AA	PCHannumAA
Not Adjusted for Cell Composition					
CSP	0.00	0.03	0.04	-0.03	-0.06*
SIP	0.31***	0.26***	0.26***	0.05	0.11***
SRP	0.25***	0.21***	0.19***	0.09***	0.15***
Sum Score	0.29***	0.25***	0.24***	0.07*	0.12***
SenMayo	0.24***	0.21***	0.16***	0.09***	0.15***
Adjusted for Cell Composition					
CSP	0.07***	0.06	0.09***	0.00	-0.02
SIP	0.09***	0.11***	0.13***	0.00	-0.02
SRP	0.05*	0.08**	0.05*	0.06*	0.05*
Sum Score	0.09***	0.12***	0.11***	0.04	0.02
SenMayo	0.01	0.05	-0.01	0.06*	0.05

Note

* p<0.05, ** p<0.01, *** p<0.001

CSP – Canonical Senescence Pathway Score; SIP – Senescence Initiating Pathway Score; SRP – Senescence Response Pathway Score; Sum Score – Senescence Summary Score; SenMayo – SenMayo Score; ExpBioAge – Expanded Biological Age; AA – Age Acceleration; PC – Principal Component.

All models are adjusted for all covariates (age, sex, race/ethnicity, education, BMI categories, smoking, drinking, insomnia symptoms), and batch/plate. For the models adjusted for cell composition, the percentages of granulocytes, natural killer cells, B cells, CD4 cells, CD8 cells, and monocytes are included in the models as covariates. Standardized coefficients are reported.

Supplementary Table 13. Five-Fold Cross-Validation Results:

Outcome	Predictor	CV R2	Main Model R2	RMSE	MAE
PCGrimAgeAA	CSP	0.31	0.35	3.26	2.53
PCGrimAgeAA	SIP	0.37	0.41	3.12	2.43
PCGrimAgeAA	SRP	0.35	0.40	3.17	2.46
PCGrimAgeAA	Sum Score	0.36	0.40	3.15	2.45
PCGrimAgeAA	SenMayo	0.35	0.39	3.17	2.46
PhenoAgeAA	CSP	0.01	0.04	6.82	5.22
PhenoAgeAA	SIP	0.04	0.08	6.68	5.10
PhenoAgeAA	SRP	0.04	0.07	6.73	5.14
PhenoAgeAA	Sum Score	0.04	0.08	6.70	5.11
PhenoAgeAA	SenMayo	0.04	0.07	6.72	5.12
DunedinPACE	CSP	0.18	0.22	0.13	0.10
DunedinPACE	SIP	0.22	0.26	0.13	0.10
DunedinPACE	SRP	0.20	0.25	0.13	0.10
DunedinPACE	Sum Score	0.21	0.26	0.13	0.10
DunedinPACE	SenMayo	0.19	0.24	0.13	0.10
ExpBioAgeAA	CSP	0.05	0.09	8.31	6.08
ExpBioAgeAA	SIP	0.08	0.12	8.16	6.01
ExpBioAgeAA	SRP	0.07	0.11	8.22	6.04
ExpBioAgeAA	Sum Score	0.07	0.11	8.18	6.03
ExpBioAgeAA	SenMayo	0.05	0.10	8.29	6.10
Multimorbidity	CSP	0.09	0.13	0.96	0.76
Multimorbidity	SIP	0.11	0.14	0.95	0.75
Multimorbidity	SRP	0.10	0.14	0.95	0.76
Multimorbidity	Sum Score	0.11	0.14	0.95	0.75
Multimorbidity	SenMayo	0.10	0.13	0.95	0.75
Cognitive Function	CSP	0.26	0.29	3.79	2.99
Cognitive Function	SIP	0.26	0.29	3.78	2.98
Cognitive Function	SRP	0.26	0.29	3.79	2.99
Cognitive Function	Sum Score	0.26	0.29	3.79	2.99
Cognitive Function	SenMayo	0.26	0.29	3.79	2.99
6yrMortality	CSP	0.20	0.24	0.52	0.27
6yrMortality	SIP	0.15	0.26	0.47	0.22
6yrMortality	SRP	0.05	0.25	0.46	0.21
6yrMortality	Sum Score	0.12	0.25	0.50	0.25
6yrMortality	SenMayo	0.22	0.25	0.50	0.26

Note

Cross-validation (CV) R2, RMSE, and MAE values in the table are the average values across the 5 folds.

For logistic regression models (models using 6-year mortality as the outcome), pseudo-R-squares (McFadden's R-squares) are calculated.

Supplementary Table 14. Measurement Occasions for Data Collection

2014	2016	2022
Systolic blood pressure, peak flow, and HbA1c for a random half of the HRS biomarker sample (Used to compute ExpBioAge)	Systolic blood pressure, peak flow, and HbA1c for a random half of the HRS biomarker sample (Used to compute ExpBioAge)	Vital status report (used to code 6-year mortality)
Height and weight for a random half of the HRS biomarker sample (Used to calculate BMI). When physical measures are not available/missing, self-reported height and weight on 2016 are used to calculate BMI.	19 clinical-level venous-blood-based biomarkers (Used to compute ExpBioAge)	
	Health outcomes, including cognitive function and multimorbidity	
	Epigenetic aging measures (PC GrimAge AA, PhenoAge, DunedinPACE) computed from venous-blood-based DNA methylation data	
	Gene expression composite scores (CSP, SIP, SRP, senescence summary, and SenMayo scores) computed from venous-blood-based RNA sequencing data	
	Height and weight for a random half of the HRS biomarker sample (Used to calculate BMI). When physical measures are not available/missing, self-reported height and weight on 2016 are used to calculate BMI.	
	All other covariates	

Supplementary Table 15. The Gene Lists for Gene Expression Composite Scores

CSP Genes	SIP Genes	SRP Genes	SenMayo Genes (1)	SenMayo Genes (2)
ATM	*ABL1*	*AKT1*	*ACVR1B*	*IGFBP5*
BMI1	*AKT1*	*CALR*	*ANG*	*IGFBP6*
CCND1	*ALDH1A3*	*CCL2*	*ANGPT1*	*IGFBP7*
CCNE1	*CCNA2*	*CCL20*	*ANGPTL4*	*IL10*
CDK2	*CCNB1*	*CCL5*	*AREG*	*IL13*
CDK4	*CDC25C*	*CCL8*	*AXL*	*IL15*
CDK6	*CDKN1B*	*CD44*	*BEX3*	*IL18*
CDKN1A	*CDKN1C*	*COL1A1*	*BMP2*	*IL1A*
CDKN2A	*CDKN2B*	*COL3A1*	*BMP6*	*IL1B*
CDKN2D	*CDKN2C*	*CXCL1*	*C3*	*IL2*
CHEK1	*CITED2*	*CXCL2*	*CCL1*	*IL32*
CHEK2	*CREG1*	*CXCL5*	*CCL13*	*IL6*
E2F1	*EGR1*	*CXCL8*	*CCL16*	*IL6ST*
E2F3	*GADD45A*	*FGF2*	*CCL2*	*IL7*
ETS1	*GSK3B*	*FGF7*	*CCL20*	*INHA*
ETS2	*HRAS*	*FN1*	*CCL24*	*IQGAP2*
MDM2	*ID1*	*GLB1*	*CCL26*	*ITGA2*
RB1	*IFNG*	*IGFBP2*	*CCL3*	*ITPKA*
RBL2	*IGF1*	*IGFBP4*	*CCL3L1*	*JUN*
SATB1	*IGF1R*	*IGFBP7*	*CCL4*	*KITLG*
TP53	*IGFBP3*	*IL11*	*CCL5*	*LCP1*
TWIST1	*IGFBP5*	*IL1A*	*CCL7*	*MIF*
-	*IGFBP7*	*IL1B*	*CCL8*	*MMP1*
-	*ING1*	*IL6*	*CD55*	*MMP10*
-	*IRF3*	*LMNB1*	*CD9*	*MMP12*
-	*IRF5*	*MMP1*	*CSF1*	*MMP13*
-	*IRF7*	*MMP10*	*CSF2*	*MMP14*
-	*MAP2K1*	*MMP12*	*CSF2RB*	*MMP2*
-	*MAP2K3*	*MMP3*	*CST4*	*MMP3*
-	*MAP2K6*	*NFIA*	*CTNNB1*	*MMP9*
-	*MAPK14*	*PLAUR*	*CTSB*	*NAP1L4*
-	*MORC3*	*PTEN*	*CXCL1*	*NRG1*
-	*MYC*	*SERPINB2*	*CXCL10*	*PAPPA*
-	*NBN*	*SERPINE1*	*CXCL12*	*PECAM1*
-	*NFKB1*	*SPARC*	*CXCL16*	*PGF*
-	*NOX4*	*TBX2*	*CXCL2*	*PIGF*
-	*PCNA*	*TBX3*	*CXCL3*	*PLAT*
-	*PIK3CA*	*TGFB1I1*	*CXCL8*	*PLAU*
-	*PLAU*	*TIMP1*	*CXCR2*	*PLAUR*
-	*PRKCD*	*TIMP2*	*DKK1*	*PTBP1*
-	*RBL1*	*TNF*	*EDN1*	*PTGER2*
-	*SIRT1*	*VEGFA*	*EGF*	*PTGES*
-	*SOD1*	*VEGFB*	*EGFR*	*RPS6KA5*
-	*SOD2*	*VIM*	*EREG*	*SCAMP4*
-	*TERF2*	-	*ESM1*	*SELPLG*
-	*TERT*	-	*ETS2*	*SEMA3F*
-	*TGFB1*	-	*FAS*	*SERPINB4*
-	*TP53BP1*	-	*FGF1*	*SERPINE1*
-	-	-	*FGF2*	*SERPINE2*
-	-	-	*FGF7*	*SPP1*
-	-	-	*GDF15*	*SPX*
-	-	-	*GEM*	*TIMP2*
-	-	-	*GMFG*	*TNF*
-	-	-	*HGF*	*TNFRSF10C*

-	-	-	*HMGB1*	*TNFRSF11B*
-	-	-	*ICAM1*	*TNFRSF1A*
-	-	-	*ICAM3*	*TNFRSF1B*
-	-	-	*IGF1*	*TUBGCP2*
-	-	-	*IGFBP1*	*VEGFA*
-	-	-	*IGFBP2*	*VEGFC*
-	-	-	*IGFBP3*	*VGF*
-	-	-	*IGFBP4*	*WNT16*
-	-	-	-	*WNT2*

Note
CSP – Canonical Senescence Pathway Score; SIP – Senescence Initiating Pathway Score; SRP – Senescence Response Pathway Score

ScoreDehkordi SK, Walker J, Sah E, Bennett E, Atrian F, Frost B, Woost B, Bennett RE, Orr TC, Zhou Y, Andhey PS. Profiling senescent cells in human brains reveals neurons with CDKN2D/p19 and tau neuropathology. *Nature aging*. 2021 Dec;1(12):1107-16. https://doi.org/10.1038/s43587-021-00142-3

Saul D, Kosinsky RL, Atkinson EJ, Doolittle ML, Zhang X, LeBrasseur NK, Pignolo RJ, Robbins PD, Niedernhofer LJ, Ikeno Y, Jurk D. A new gene set identifies senescent cells and predicts senescence-associated pathways across tissues. *Nature communications*. 2022 Aug 16;13(1):4827. https://doi.org/10.1038/s41467-022-32552-1

Supplementary Figure 1. Correlation between Brain and Blood Expression of Genes Included in Dehkordi et al. Lists

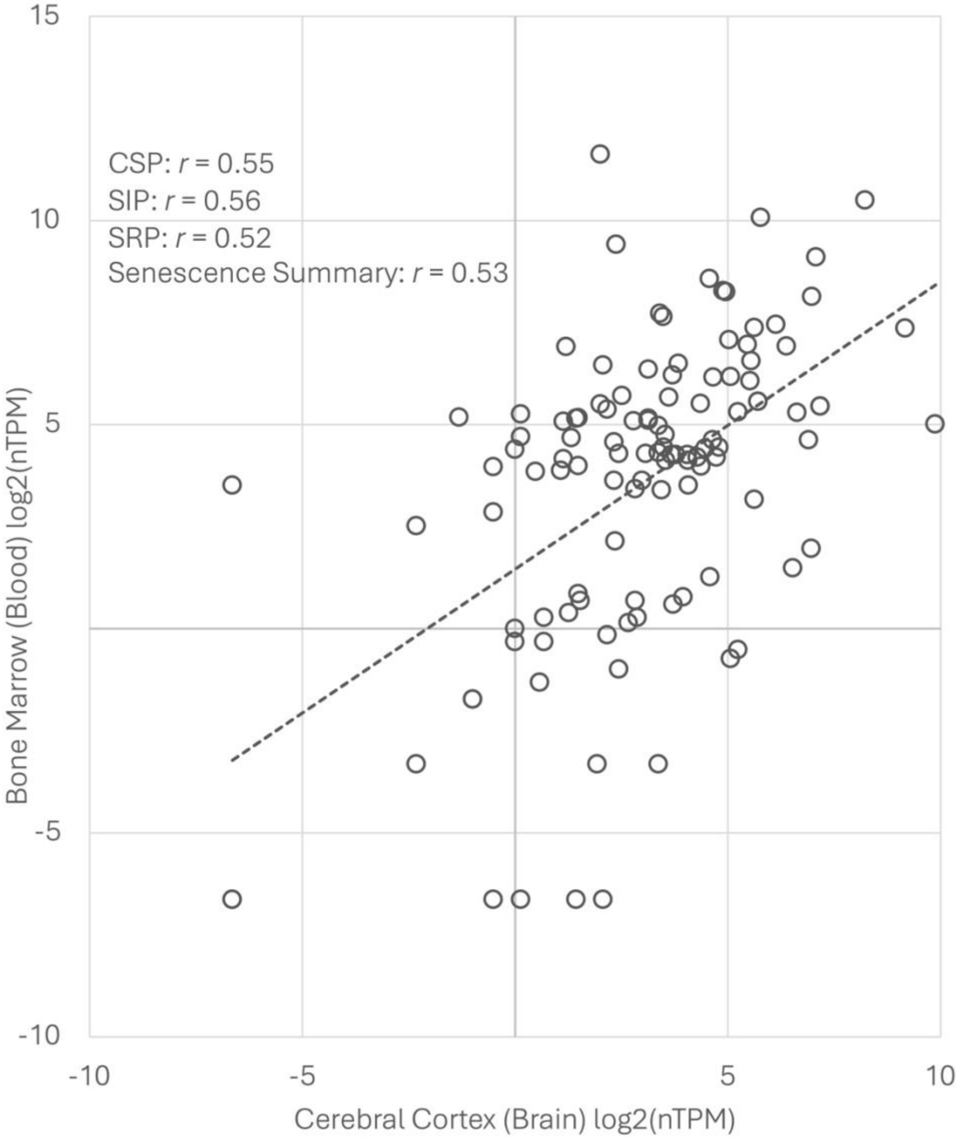

Note
CSP – Canonical Senescence Pathway Score; SIP – Senescence Initiating Pathway Score; SRP – Senescence Response Pathway Score; Senescence Summary – Senescence Summary Score.
Expression levels are indicated by log2 transformed normalized expression (nTPM) values derived from information on the open-access online source Human Protein Atlas (https://www.proteinatlas.org/), which documents the RNA expression of each gene across different organs. Specifically, the expression level in the brain is based on cerebral cortex, and the expression level in blood is based on bone marrow. Pearson correlation coefficients (*r*) are reported.
