# Supplementary material for: Gene expression composite scores of cellular senescence predict aging health outcomes in the Health and Retirement Study
Source: Nat Commun. 2025 Oct 10;16:9044. doi: 10.1038/s41467-025-64835-8 (PMC12514264; doi:10.1038/s41467-025-64835-8)
Supplement: Supplementary file 3 — Reporting Summary [file 41467_2025_64835_MOESM3_ESM.pdf]

nature portfolio

Corresponding author(s): Qiao Wu and Eileen Crimmins

Last updated by author(s): Sep 12, 2025

Reporting Summary

Nature Portfolio wishes to improve the reproducibility of the work that we publish. This form provides structure for consistency and transparency in reporting. For further information on Nature Portfolio policies, see our Editorial Policies and the Editorial Policy Checklist.

Statistics

For all statistical analyses, confirm that the following items are present in the figure legend, table legend, main text, or Methods section.

n/a	Confirmed	
☒	☐	The exact sample size (*n*) for each experimental group/condition, given as a discrete number and unit of measurement
☒	☐	A statement on whether measurements were taken from distinct samples or whether the same sample was measured repeatedly
☐	☒	The statistical test(s) used AND whether they are one- or two-sided<br>*Only common tests should be described solely by name; describe more complex techniques in the Methods section.*
☐	☒	A description of all covariates tested
☐	☒	A description of any assumptions or corrections, such as tests of normality and adjustment for multiple comparisons
☐	☒	A full description of the statistical parameters including central tendency (e.g. means) or other basic estimates (e.g. regression coefficient) AND variation (e.g. standard deviation) or associated estimates of uncertainty (e.g. confidence intervals)
☐	☒	For null hypothesis testing, the test statistic (e.g. *F*, *t*, *r*) with confidence intervals, effect sizes, degrees of freedom and *P* value noted<br>*Give P values as exact values whenever suitable.*
☒	☐	For Bayesian analysis, information on the choice of priors and Markov chain Monte Carlo settings
☒	☐	For hierarchical and complex designs, identification of the appropriate level for tests and full reporting of outcomes
☐	☒	Estimates of effect sizes (e.g. Cohen's *d*, Pearson's *r*), indicating how they were calculated

Our web collection on statistics for biologists contains articles on many of the points above.

Software and code

Policy information about availability of computer code

Data collection	N/A (The current study uses secondary data)
Data analysis	Stata version 18

For manuscripts utilizing custom algorithms or software that are central to the research but not yet described in published literature, software must be made available to editors and reviewers. We strongly encourage code deposition in a community repository (e.g. GitHub). See the Nature Portfolio guidelines for submitting code & software for further information.

Data

Policy information about availability of data

All manuscripts must include a data availability statement. This statement should provide the following information, where applicable:

- Accession codes, unique identifiers, or web links for publicly available datasets
- A description of any restrictions on data availability
- For clinical datasets or third party data, please ensure that the statement adheres to our policy

Much of the Health and Retirement Study (HRS) data can be accessed via the University of Michigan site (https://hrs.isr.umich.edu/data-products). Sensitive health data from HRS is only available under restricted access. Venous Blood Study (VBS) and Biomarker Data used in the current study can be accessed upon request (https://hrsdata.isr.umich.edu/data-products/sensitive-health). Genetic and transcriptomic data from HRS used in the current study can be accessed through NIAGADS upon application (https://hrs.isr.umich.edu/data-products/genetic-data).

Research involving human participants, their data, or biological material

Policy information about studies with human participants or human data. See also policy information about sex, gender (identity/presentation), and sexual orientation and race, ethnicity and racism.

Reporting on sex and gender	The current study used the term sex to represent biological attribute. The findings were not sex-specific. Sex was determined based on self-reporting, as described in the method section. No sex-specific analyses were performed. The association between sex and cellular senescence was assessed. How sex structure of the sample might affect the findings was carefully discussed in the discussion section.
Reporting on race, ethnicity, or other socially relevant groupings	Race/ethnicity was used to understand the sociodemographic and behavioral pattern of senescence scores. Racial/ethnic groups include non-Hispanic White, non-Hispanic Black, Hispanic, and non-Hispanic Others.
Population characteristics	Our study sample comes from the Health and Retirement Study (HRS), a nationally representative longitudinal study of US adults older than age 50. In 2016, a representative subsample of HRS Venous Blood Sample (VBS) participants was selected for innovative assays reflecting cellular-/molecular-level mechanisms of aging, including RNA sequencing. After applying survey weight, our sample represents community-dwelling Americans older than age 50 in terms of the distributions of demographic and socioeconomic characteristics.
Recruitment	All panel respondents who completed an HRS interview during the 2016 wave were asked to consent to a venous blood draw with the exception of proxy respondents and nursing home residents. The request was made by their HRS interviewer at the end of the interview (telephone and in person modes), with the offer of a fifty-dollar incentive payment to be sent by check. That incentive amount and procedure is consistent with other voluntary ancillary activities in HRS where we routinely pay in advance of completion. No higher amounts were offered. More information on this secondary data source can be found: https://hrsdata.isr.umich.edu/data-products/2016-venous-blood-study-vbs
Ethics oversight	This secondary data analysis received IRB approval (UP-18-00229) from the Human Research Protection Program, University of Southern California.

Note that full information on the approval of the study protocol must also be provided in the manuscript.

Field-specific reporting

Please select the one below that is the best fit for your research. If you are not sure, read the appropriate sections before making your selection.

☐ Life sciences ☒ Behavioural & social sciences ☐ Ecological, evolutionary & environmental sciences

For a reference copy of the document with all sections, see nature.com/documents/nr-reporting-summary-flat.pdf

Behavioural & social sciences study design

All studies must disclose on these points even when the disclosure is negative.

Study description	Quantitative cross-sectional secondary data analysis
Research sample	We used the Venous Blood Sample of the Health and Retirement Study (https://hrs.isr.umich.edu/). Our sample is representative of community-dwelling Americans older than age 50.
Sampling strategy	Health and Retirement Study (HRS) is a longitudinal panel study that surveys a representative sample of approximately 20,000 people in America. More information on sampling strategy: https://hrs.isr.umich.edu/documentation/survey-design
Data collection	HRS core survey data were collected through interview. Anthropometric markers were collected through the enhanced-face-to-face interview. Biomarkers data were based on venous blood collection.
Timing	We used the data from the 2016 survey wave of HRS. For Systolic blood pressure, peak flow, HbA1c, weight, and height, data from a random half of the sample were collected in 2014. Data from the other half of the sample were collected in 2016. We used the vital status report in 2022 to code the 6-year mortality variable.
Data exclusions	After the quality control process, a total of 3738 respondents have valid gene expression values. All participants with RNA data also have valid epigenetic aging measures based on DNA methylation. Our main analytical sample consists of 3580 respondents who further have complete information on sociodemographic characteristics, behavioral factors, and multimorbidity. The models using 6-year mortality as the outcome are based on a subsample of 3554 respondents who have non-missing vital status at the 2022 follow-up. The models using ExpBioAge as the outcome are based on a subsample of 2660 respondents who further have non-missing corresponding biomarkers.
Non-participation	Participation by respondents to the blood collection protocol in 2016 was excellent, despite the need for a two-stage consent process which experience has shown to be a negative for response rates. Our final VBS sample with collected data was n=9934. The consent rate was 78.5% for cases interviewed through September 15, 2017 and of these, 82.9% had a completed collection for a final

completion rate of 65% among eligible cases. More information: https://hrsdata.isr.umich.edu/data-products/2016-venous-blood-study-vbs

Randomization	N/A

Reporting for specific materials, systems and methods

We require information from authors about some types of materials, experimental systems and methods used in many studies. Here, indicate whether each material, system or method listed is relevant to your study. If you are not sure if a list item applies to your research, read the appropriate section before selecting a response.

Materials & experimental systems

n/a	Involved in the study
☒	☐ Antibodies
☒	☐ Eukaryotic cell lines
☒	☐ Palaeontology and archaeology
☒	☐ Animals and other organisms
☒	☐ Clinical data
☒	☐ Dual use research of concern
☒	☐ Plants

Methods

n/a	Involved in the study
☒	☐ ChIP-seq
☒	☐ Flow cytometry
☒	☐ MRI-based neuroimaging

Plants

Seed stocks	*Report on the source of all seed stocks or other plant material used. If applicable, state the seed stock centre and catalogue number. If plant specimens were collected from the field, describe the collection location, date and sampling procedures.*
Novel plant genotypes	*Describe the methods by which all novel plant genotypes were produced. This includes those generated by transgenic approaches, gene editing, chemical/radiation-based mutagenesis and hybridization. For transgenic lines, describe the transformation method, the number of independent lines analyzed and the generation upon which experiments were performed. For gene-edited lines, describe the editor used, the endogenous sequence targeted for editing, the targeting guide RNA sequence (if applicable) and how the editor was applied.*
Authentication	*Describe any authentication procedures for each seed stock used or novel genotype generated. Describe any experiments used to assess the effect of a mutation and, where applicable, how potential secondary effects (e.g. second site T-DNA insertions, mosiacism, off-target gene editing) were examined.*
